# Effects of CeO_2_ Nanoparticles on Nutritional Quality of Two Crop Plants, Corn (*Zea mays* L.) and Soybean (*Glycine max* L.)

**DOI:** 10.3390/molecules28041798

**Published:** 2023-02-14

**Authors:** Xin Gui, Chaonan Dong, Shixian Fan, Chunlei Jiao, Zhuda Song, Jiaqi Shen, Yong Zhao, Xuanzhen Li, Fawen Zhang, Yuhui Ma, Xiao He, Aijun Lin, Zhiyong Zhang

**Affiliations:** 1 Key Laboratory for Biomedical Effects of Nanomaterials and Nanosafety, Institute of High Energy Physics, Chinese Academy of Sciences, Beijing 100049, China; 2 College of Forestry, Henan Agriculture University, Zhengzhou 450002, China; 3 Department of Environmental Science and Engineering, Beijing University of Chemical Technology, Beijing 100029, China; 4 School of Nuclear Science and Technology, University of Chinese Academy of Sciences, Beijing 100049, China

**Keywords:** cerium dioxide, nanoparticles, corn, soybean, nutritional quality

## Abstract

With the widespread applications of manufactured nanoparticles (NPs), there are increasing concerns about their potential adverse effects on the environment and living systems. Many studies demonstrated that NPs could significantly affect the growth and development of crop plants. However, knowledge regarding the impacts of NPs on crop quality is rather limited. In this study, the effects of CeO_2_ NPs (25, 75, and 225 mg Ce/kg) and CeCl_3_ (25 mg Ce/kg) on the nutritional components of soil-cultivated corn and soybean plants were evaluated. Both treatments tended to decrease the dry weight of grain per plant, while only 225 mg/kg CeO_2_ NPs on soybean and CeCl_3_ on corn showed statistical significance compared with the respective control. CeO_2_ NPs at 225 mg/kg significantly decreased the content of starch in the corn kernels by 18.2% but increased total phenols in soybean seeds by 18.4%. Neither CeO_2_ NPs nor CeCl_3_ significantly affected the contents of minerals in corn kernels except for Zn. However, in the case of soybean, the two treatments tended to decrease the contents of P, Zn, Mn, and Mo but increase the content of S. Overall, the results suggest that CeO_2_ NPs and Ce^3+^ ions showed similar but not identical effects on corn and soybean plants. CeO_2_ NPs affect the nutritional quality of crop plants in a species-dependent manner.

## 1. Introduction

CeO_2_ NPs are one of the most applied engineered NPs, and they probably end up in landfills and agricultural soils [1]. The increasing abundance and availability of CeO_2_ NPs in the terrestrial environment have led to concerns regarding their potential impacts on soil organisms, especially edible crop plants. Previous studies have found that exposure to CeO_2_ NPs can impede growth and affect the nutritional quality of crop plants. Rico et al. reported that CeO_2_ NPs at 500 mg/kg significantly changed the nutritive value of rice grains by reducing the contents of Fe, S, prolamin, glutelin, lauric and valeric acids, and starch [2]. Zhao et al. evaluated the accumulation of Ce, as well as the nutrient concentrations and distribution of corn kernels after CeO_2_ NP treatments [3]. CeO_2_ NPs at 800 mg/kg reduced the crop yield by 38% and changed the allocation of calcium in kernels. Marchiol et al. found that CeO_2_ NPs at 500 and 1000 mg/kg extended the growth cycle of barley and reduced the number of tillers and spikes per plant [4]. Pošćić et al. also demonstrated that barely kernels were negatively affected by CeO_2_ NPs at 500 and 1000 mg/kg, which did not affect β-glucans but reduced amylose content. K, S, and B concentrations in kernels were significantly reduced by CeO_2_ NPs [5]. Rico et al. reported that barley plants exposed to CeO_2_ NPs at 500 mg/kg did not form grains, while CeO_2_ NPs at 125 mg/kg enhanced the amino acids contents and mineral concentrations in grain [6]. However, other authors also reported positive effects of CeO_2_ NPs on crop plants. Bradfield et al. observed that CeO_2_ NPs at 500 and 1000 mg/kg had no adverse effect on sweet potato yield, but slightly increased the tuber diameter [7]. Du et al. found in a field investigation that CeO_2_ NPs at 100 and 400 mg/kg showed no effects on the final yield but increased the grain protein by 24.8% and 32.6% [8]. In general, current knowledge on effect of CeO_2_ NPs on food crops is still scarce. Exposure concentrations of CeO_2_ NPs used in most investigations are too high and not relevant to the environment.

The main objective of this study was to investigate whether the treatments of CeO_2_ NPs at relatively low concentrations could affect the nutritional quality of two agriculturally significant crop plants, corn, and soybean. As major agricultural crops, corn and soybean are planted in most of the farming areas in the world and are two of the main sources of plant-based proteins [9]. In the present study, corn and soybean seeds were germinated and grown to full maturity in soil amended with CeO_2_ NPs at 0, 25, 75, and 225 mg of Ce/kg. CeCl_3_ was used as the ionic control for CeO_2_ NPs. Nutritional compositions of corn kernels and soybean seeds were analyzed by using several biochemical and spectroscopic techniques. The results provide important information for understanding the potential risks of CeO_2_ NPs to food safety and human health.

## 2. Results

### 2.1. Dry Weight of Grain

**Figure 1:**
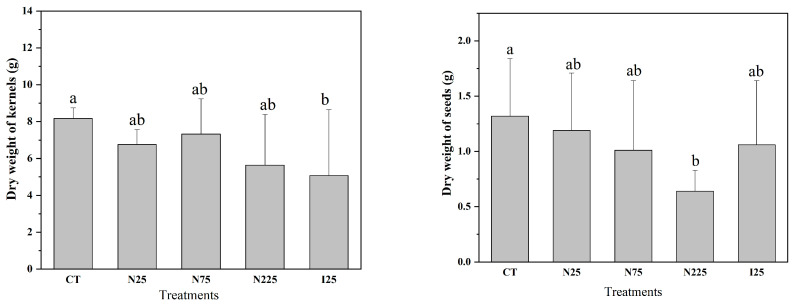
Dry weight of grain per plant of corn (**Left**) and soybean (**Right**). CT: control; N: CeO_2_ NPs; I: CeCl_3_. Different letters stand for statistical differences between treatments at *p* ≤ 0.05.

The dry weights of corn kernels and soybean seeds are shown in Figure 1. CeO_2_ NPs at 25, 75, and 225 mg/kg decreased the dry weight of grain per plant of corn and soybean by 17.3%, 10.3%, 31.0, and 9.85%, 23.5%, 51.5%, respectively, compared to the control, but only the difference on soybean at the highest concentration was statistically significant. Ce^3+^ at 25 mg/kg decreased the dry weight of grain per plant of corn and soybean by 37.9% and 19.7%, respectively, compared to the control, but only the difference in corn was statistically significant. A nested two-factor analysis of variance showed that the dry weight of soybean seeds and corn kernels was significantly different under the same treatment.

### 2.2. Organic Nutrients

**Figure 2:**
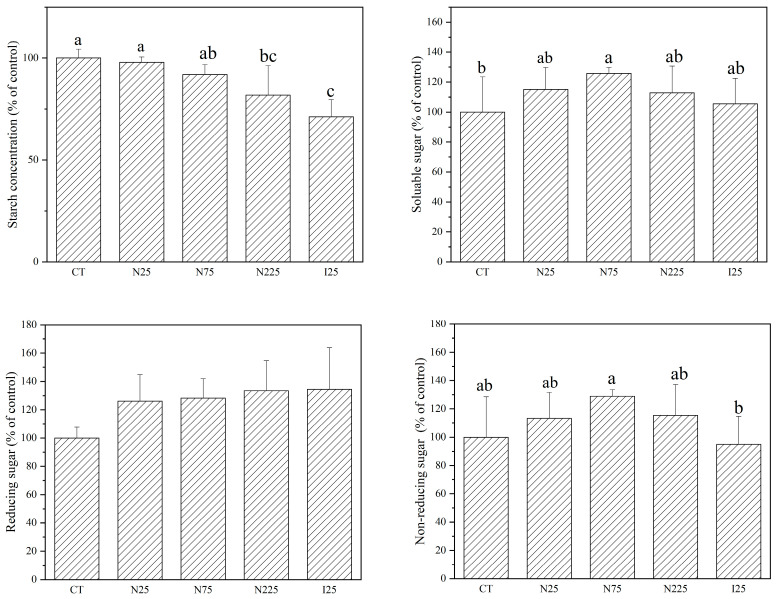
Contents of starch, soluble sugar, reducing sugar, and non-reducing sugar in the corn kernels harvested from plants cultivated in CeO_2_ NPs/CeCl_3_-amended soil. CT: control; N: CeO_2_ NPs; I: CeCl_3_. Different letters stand for statistical differences between treatments at *p* ≤ 0.05.

As shown in Figure 2, the effects of CeO_2_ NP or Ce^3+^ ion treatments showed a tendency to decrease the contents of starch but increase the contents of soluble sugar, reducing sugar and non-reducing sugar in corn kernels. However, only CeO_2_ NPs at 225 mg/kg and Ce^3+^ ions (25 mg/kg) significantly reduced the starch contents by 18.2% and 28.7%. CeO_2_ NPs at 75 mg/kg significantly elevated the soluble sugar content by 29.5% in comparison with the control. There were no significant differences in soluble protein, proline, and total phenol contents in the corn kernels after CeO_2_ NP or Ce^3+^ ion treatments with respect to the control (Figure S1).

**Figure 3:**
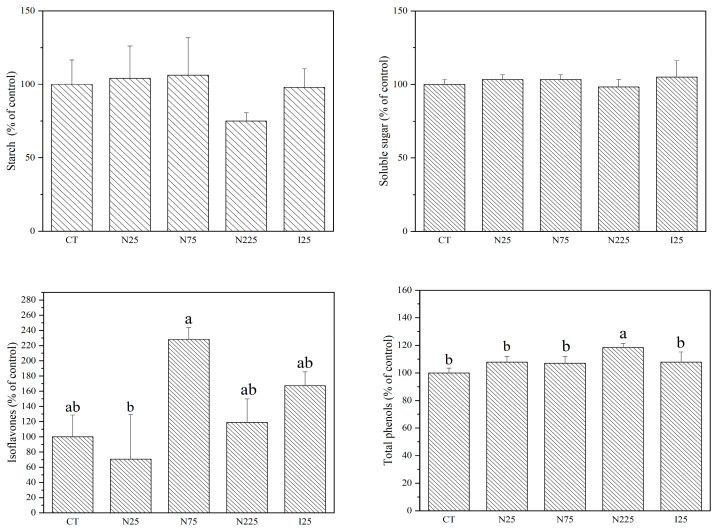
Contents of starch, soluble sugar, isoflavones, and total phenols in the soybean seeds harvested from plants cultivated in CeO_2_ NPs/CeCl_3_-amended soil. CT: control; N: CeO_2_ NPs; I: CeCl_3_. Different letters stand for statistical differences between treatments at *p* ≤ 0.05.

As for soybean, the contents of starch, soluble sugar (Figure 3), reducing sugar, soluble protein, lysine, fat, and vitamin E (Figure S2) were not found to be significantly affected by CeO_2_ NP treatments at all concentrations. Neither CeO_2_ NPs nor Ce^3+^ ions significantly affected the contents of isoflavones as compared to the control. However, the plants that were treated with 75 mg/kg CeO_2_ NPs had significantly higher isoflavone contents in the seeds than those treated with CeO_2_ NP at 25 mg/kg. CeO_2_ NPs at 225 mg/kg significantly increased the content of total phenols by 18.4% in comparison with the control (Figure 3). Ce^3+^ ions showed no significant effect on the eight organic nutrients except reducing sugar, which was significantly decreased by 46.3% in comparison with the control (Figure S2). A nested two-factor analysis of variance showed that the organic nutrients between soybean seeds and corn kernels were significantly different under the same treatment.

### 2.3. Contents of Mineral Elements

**Figure 4:**
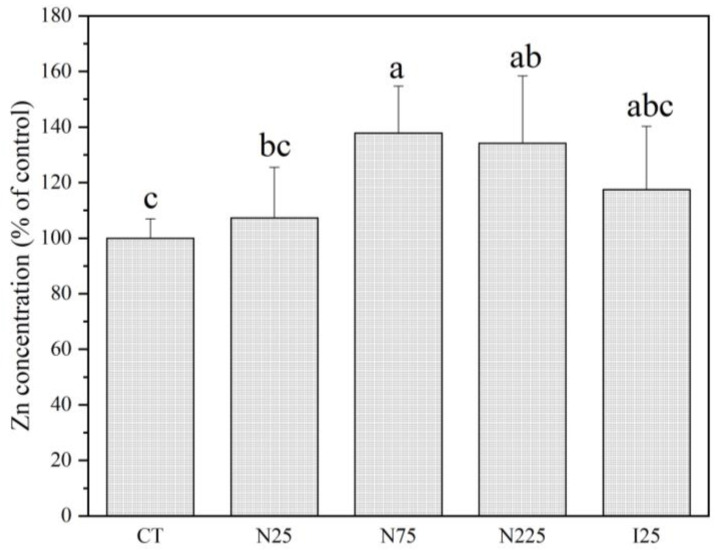
Contents of Zn in the corn kernels harvested from plants cultivated in CeO_2_ NPs/CeCl_3_-amended soil. CT: control; N: CeO_2_ NPs; I: CeCl_3_. Different letters stand for statistical differences between treatments at *p* ≤ 0.05.

**Figure 5:**
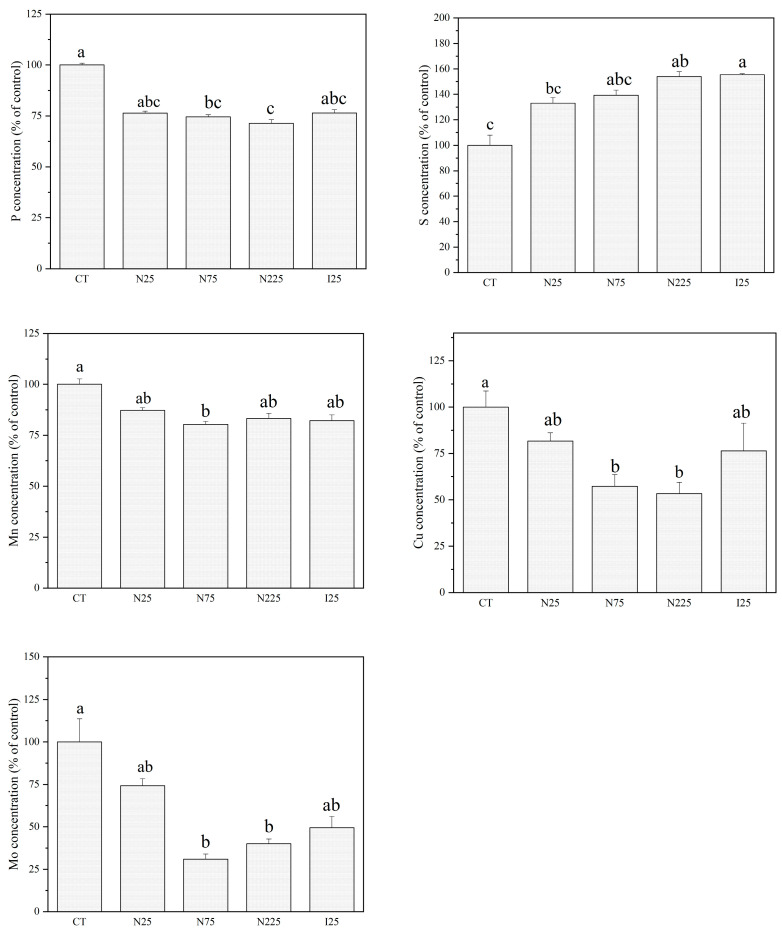
Contents of P, S, Mn, Cu, and Mo in the soybean seeds harvested from plants cultivated in CeO_2_ NPs/CeCl_3_-amended soil. CT: control; N: CeO_2_ NPs; I: CeCl_3_. Different letters stand for statistical differences between treatments at *p* ≤ 0.05.

The contents of mineral elements (Ca, P, S, Fe, Cu, Zn, Mn, and Mo) in corn kernels and soybean seeds are shown in Figure 4 and Figure 5 and Figures S3 and S4. CeO_2_ NPs and Ce^3+^ ions did not affect the contents of measured mineral nutrients in corn kernels except for Zn (Figure 4 and Figure S3). Both treatments tended to increase Zn accumulation in the kernels, while only 75 mg/kg and 225 mg/kg CeO_2_ NPs showed statistical significance with 36.2% and 32.7% increments. In the soybean seeds, CeO_2_ NPs and Ce^3+^ ions tended to decrease the contents of P, Zn, Mn, and Mo but increased the contents of S (Figure 5). At 75 and 225 mg/kg, CeO_2_ NPs significantly decrease the contents of P, Mn, and Mo by 25.5%, 42.7%, 69.0%, and 28.7%, 46.7%, and 59.9% in comparison with control, respectively. CeO_2_ NPs at 75 mg/kg significantly decrease the content of Zn by 19.8% compared with the control. CeO_2_ NPs at 225 mg/kg and Ce^3+^ at 25 mg/kg significantly increase the contents of S by 54.1% and 55.5%, respectively, compared to the control. The contents of Ca, Fe, and Zn were not found to be significantly affected by either CeO_2_ NP or Ce^3+^ ion treatments (Figure S4).

The multiple stepwise regression analysis showed that the P content significantly affected the dry weight of corn kernels, and the change of Mo element significantly affected the dry weight of soybean seeds (Table S2).

### 2.4. Principal Component Analysis

**Figure 6:**
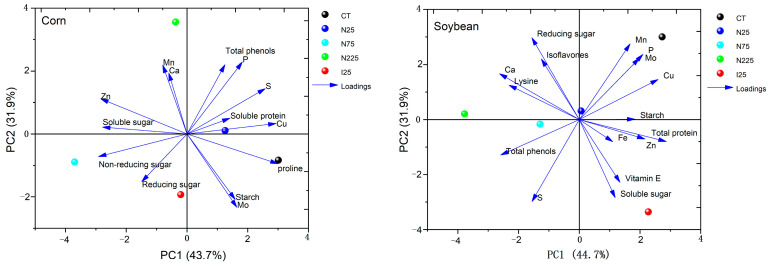
Principal component analysis plot of element content and nutritional quality indexes of maize and soybean plants among different treatments.

The distance of the line at each sample point reflects the similarity between the treatment groups; the shorter distance, the closer similarity, and vice versa (Figure 6). For corn, P, S, Cu, Zn, proline, soluble sugar, and non-soluble sugar have a large contribution to PC1. P, S, Cu, and proline show a positive correlation with PC1. Nevertheless, Zn, soluble sugar, and non-soluble sugar show a negative correlation with PC1. It is shown that the different treatments altered the nutritional profile of corn kernels in the sequence N75 > N225 > I25 > N25.

For soybean, P, Mo, Cu, Ca, soluble protein, lysine, and total phenols have a large contribution to PC1. P, Mo, Cu, and soluble protein show a large positive correlation with PC1. However, Ca, lysine, and total phenols show a negative correlation with PC1. CeO_2_ NP treatments dose-dependently affected the nutritional quality of the soybean seeds.

## 3. Discussion

Ce is the most abundant rare earth element (REE) on the Earth and has different chemical properties from the other REEs due to the existence of two stable oxidation states, +3 and +4, with the electronic configurations of [Xe]4f^1^ and [Xe]4f^0^, respectively [10]. Nano-sized CeO_2_ has oxygen vacancies and defects on the surface, which enables the reversible shift of Ce oxidation states between +3 and +4 depending on the availability of O atoms. The change of valence allows Ce NPs to store O (Ce(IV)O_2_) under oxidizing conditions and to release O (Ce_2_(III)O_3_) under reducing conditions, giving CeO_2_ NPs the ability to act as catalysts for redox reactions [11,12].

The oxidation state of Ce(IV) is generally considered more chemically stable than that of Ce(III) due to the electronic configuration [Xe]4f^0^ (Ce(IV)) being more stable as empty than [Xe]4f^1^ (Ce(III)) [13]. However, previous studies indicate that CeO_2_ NPs underwent transformation (i.e., Ce(IV)→Ce(III)) in the rhizosphere of plants with the assistance of reducing agents (e.g., exudates, microbes) [14,15]. Meanwhile, Ce(III) can be re-oxidized into Ce(IV) through an oxidation reaction by Mn oxides in soil [16]. Accordingly, chemical species of Ce in plant-soil systems depend on the microenvironment in which they are located. Both Ce(IV) and Ce(III) species coexist in the soil amended with CeO_2_ NPs [17]. We previously found that in the roots of corn plants that were treated with CeO_2_ NPs at 225 mg/kg for 90 days, 8.4% of Ce was present as Ce(III) [18]. Consequently, the observed physiological and biochemical effects of CeO_2_ NPs on terrestrial plants were the results of the joint action of Ce(IV) and Ce(III) species. It is suggested that the toxicity of CeO_2_ NPs is mostly due to the released Ce(III) species [18,19]. Considering the chemical species of Ce in plant tissues and inherent physiological differences among plants, many factors, such as culture medium, plant species, and NP properties, may affect the phytotoxicity of CeO_2_ NPs [15,19,20].

In the present study, corn and soybean plants were treated with commercial CeO_2_ NPs (25, 75, and 225 mg/kg) and Ce^3+^ ions (25 mg/kg) during the whole growth cycle. The results indicate that the nutritional profiles of corn and soybean grains were differently affected by CeO_2_ NP exposure. CeO_2_ NPs and Ce^3+^ ions showed similar but not identical effects on the tested plants, which were consistent with the previous reports [18]. Particle size is a critical parameter in the safety assessment of engineered NPs. Smaller particles generally have higher reactivity than larger ones. In a previous simulation study, we demonstrated that in the presence of reducing substances (vitamin C or catechol), smaller CeO_2_ NPs (7 nm) released more Ce^3+^ ions than the larger NPs (16 and 25 nm) [19]. It can be expected that smaller CeO_2_ NPs would show a more detrimental effect on crop plants than the larger ones.

Translocation of Ce(III), a trivalent lanthanide, in plants is difficult. As a consequence, root tissues were the main target of Ce(III) accumulation [14,18]. The exact mechanism of phyototoxicity of Ce(III) is still unclear [21]. Previous studies show that, like other trivalent lanthanide ions, Ce(III) treatments disturb the homeostasis of antioxidative systems in plant roots, affect the uptake and distribution of certain mineral nutrients, and consequently may be responsible for mineral disturbances and depression of plant growth and productivity [22,23]. We previously reported that CeO_2_ NPs at 225 mg/kg and Ce^3+^ ions at 25 mg/kg significantly decreased the contents of chlorophyll in corn leaves, thus causing a reduction of photosynthetic activity [18]. The decrease in the accumulation of assimilation products in leaves would result in a decrease in the dry weight of grain.

Starch is the primary carbohydrate in cereal grains and staple crops; it is also the raw material for deep transformation into modified starch, high-sugar syrups, alcohol, fuel, and other products [24]. Therefore, the decrease in starch contents in the corn kernels not only affected the nutritional quality of corn but also reduced its processing quality. For corn, starch is prepared and stored in “source” (leaves) during the daytime by photosynthesis, while it is hydrolyzed by amylase during the night to produce sucrose, which is transported to “sink” organs (ears and kernels) by phloem for a long distance [25,26]. Sucrose (non-reducing sugar) is the main form of photosynthetic products transported from “source” organs to “sink” organs in plants. Sucrose, as the substrate of starch, can be decomposed by sucrose invertase to produce fructose and glucose, and then the two kinds of reducing sugar can be further synthesized into starch accumulated in the grains [27,28]. In the present study, the contents of starch in the corn kernels were significantly decreased by CeO_2_ NPs at 225 mg/kg and Ce^3+^ ions at 25 mg/kg, which were accompanied by slight increases in the contents of soluble and reducing sugars. Huang et al. [29] postulated that there are certain associations between starch and soluble sugar. However, the underlying mechanisms of the changes in carbohydrate metabolism are still unknown.

Soybean is a rich source of proteins, fats, carbohydrates, dietary fiber, phytochemicals, isoflavones, phytosterols, vitamins, and minerals [30,31]. In the present study, CeO_2_ NPs at 225 mg/kg significantly increased the content of total phenols, and Ce^3+^ ions at 25 mg/kg decreased the content of reducing sugar in the soybean seeds. Both phenolic compounds and reducing sugars are involved in the responses to a number of stresses in plants [32]. Changes in the contents of total phenols and reduced sugar might be the result of oxidative stress by CeO_2_ NP and Ce^3+^ ion treatments.

Mineral elements such as Ca, P, S, Fe, Cu, Zn, Mn, and Mo are essential to human health and well-being. We previously reported that CeO_2_ NPs and Ce^3+^ ions disturbed the uptake and distribution of Ca, P, Fe, B, Zn, and Mn in corn plant tissues [18]. However, the results of the present study indicate that CeO_2_ NPs and CeCl_3_ treatments had much fewer effects on the mineral contents in the kernels than other parts of the corn plants. Only CeO_2_ at 75 and 225 mg/kg significantly elevated the contents of Zn in the kernels. These findings suggest that CeO_2_ NPs and Ce^3+^ ions modulated the elemental accumulation in the roots and leaves of corn plants without detrimental impact on the mineral contents in kernels. However, the effect of CeO_2_ NPs on the mineral element contents of soybean is notably different from that of corn. CeO_2_ NPs at 75 and 225 mg/kg significantly altered the contents of several mineral nutrients (e.g., P, Mn, and Mo). Mineral elements are delivered to different tissues after they are taken up from the roots. Plant species and their genotypes differ genetically in their ability to uptake, translocation, and accumulation of mineral elements [33,34]. The underlying mechanisms of the effect of CeO_2_ NPs on the translocation and accumulation of mineral elements in plant organs need further investigation.

## 4. Materials and Methods

### 4.1. Materials

CeO_2_ nanoparticles with an average size of about 16.3 nm were purchased from Sigma-Aldrich (CAS:1306-38-3). Physicochemical properties of the particles have been reported in a previous study [19]. CeCl_3_·7H_2_O (99.5%) was obtained from Sinopharm Chemical Reagent Co., Ltd (Shanghai, China).

### 4.2. Experimental Design and Growth Conditions

Details of the experimental design, soil, and growth conditions have been reported earlier [18]. The substrate was composed of local loam sand soil and commercial potting soil (Miracle-Gro garden soil for flowers and vegetables) with a ratio of 3:1 (*w/w*). CeO_2_ NP powders were added to 1.6 kg of the substrate to obtain final concentrations of 0 (control), 25, 75, and 225 mg Ce/kg dry soil. CeCl_3_ at 25 mg Ce/kg was used as the ionic control of CeO_2_ NPs. An early study considered 25 mg/kg CeO_2_ NPs as the environmentally relevant concentration [35]. All soils were vigorously mixed to maximize homogeneity, then placed in 5 L polypropylene containers and allowed to equilibrate for 2 weeks before the experiment started. There were six replicates in each treatment.

Corn seeds (Meizhen 204) were purchased from Beijing Baofeng Seed Co., Ltd., China, and soybean seeds (Zhonghuang 13) were obtained from Chinese Academy of Agricultural Sciences. The seeds were soaked in distilled water in the dark for 24 h. After soaking, seeds of similar size were selected and randomly planted, five seeds per pot. Irrigation was carried out every 24 h with deionized water to reach a total of 125 mL of water per kg soil throughout the period of the experiment. Corn and soybean seedlings were thinned to one plant per pot three and four weeks after sowing, respectively. The plants were grown in a greenhouse to their physiological maturity.

### 4.3. Nutritional Quality Assessment

Corn ears and soybean pods were collected at 90 and 96 days after sowing, respectively. Corn ears were separated into cobs and kernels, and soybean pods were shelled by hand. Corn kernels and soybean seeds were freeze-dried, then ground to powder. Contents of starch, soluble sugar, reducing sugar, soluble protein, proline, and total phenols in the corn kernels, and starch, soluble sugar, reducing sugar, soluble protein, fat, isoflavones, total phenols, vitamin E, and lysine in the soybean seeds, were analyzed, respectively. The seed powders of soybean were extracted in a Soxhlet extractor using petroleum ether as the solvent for fat analysis. Starch, soluble sugar, soluble protein, proline, vitamin E, and total phenol contents were quantified using commercial kits from Nanjing Jiancheng Bioengineering Institute (Nanjing, China). Soluble protein in the seeds was determined using a BCA protein assay kit (Beyotime Institute of Biotechnology, Shanghai, China). The contents of lysine and reducing sugar were quantified using commercial assay kits from Suzhou Comin Biotechnology Co., Ltd., China. The content of soybean isoflavone was measured using a commercial enzyme-linked immunosorbent assay (ELISA) kit of Shanghai Jianglai Industrial Co., Ltd., China. All tests were running following suppliers’ guidelines.

### 4.4. Quantification of Mineral Elements

Powdered seeds were digested with a mixture of plasma pure HNO_3_ and H_2_O_2_ (*v/v*: 4:1) on a heating plate. Inductively coupled plasma-mass spectrometry (ICP-MS, Thermo Elemental X7) was used for the analysis of Mn, Cu, Zn, and Mo. While macronutrient contents (P, S, K, Ca, and Fe) were determined by inductively coupled plasma–optical emission spectroscopy (ICP-OES, Perkin Elmer). Specific analytical procedures are depicted in the previous study [36]. Standard reference materials GBW07602 and GBW07603 (bush branches and leaves) were also digested and used to validate the digestion and analytical method. The obtained recoveries for all elements were between 81.1% and 127% (Table S1).

### 4.5. Data Analysis and Statistics

All statistical analyses were processed with Statistical Packages for the Social Science 22.0 (SPSS). A basic exploratory analysis of the mineral content and nutrient quality was performed using principal component analysis (PCA). To investigate the effect of the mineral element contents on the dry weight of grain per plant, we performed multiple stepwise regression analysis at a significance level of 0.05. The following indices were used as parameters for determining right variant: coefficient of determination (R2), adjusted coefficient determination (R^2^-adj), variance inflation factor (VIF), and Durbin–Waston (d). Analysis of variance (ANOVA) was used to assess the effects of crop varieties and nanoparticles or ion treatments on dry weight and nutritional quality of corn and soybeans. Statistical significant difference among treatments was performed by conducting One-way ANOVA analysis followed by LSD post hoc test. Differences among treatments were evaluated using Kruskal–Wallis H ANOVA with Mann–Whitney U test. All data were given as mean ± standard deviation (*n* = 6), and *p* < 0.05 was considered statistical significance of difference.

## 5. Conclusions

The present study systematically evaluates the effects of CeO_2_ NPs and Ce^3+^ ions on the nutritional quality of corn and soybean. CeO_2_ NPs showed no adverse effects either on the dry weight of grain per plant or on the contents of organic and mineral nutrients at an environmentally relevant concentration (25 mg/kg). However, at higher concentrations (75 and 225 mg/kg), CeO_2_ NPs significantly changed the nutritional quality of corn kernels and soybean seeds in a species-dependent manner. The effect on the nutritional composition of corn kernels is mainly in organic components, while that of soybean seeds is mainly in mineral elements. This study provides important information for a better understanding of the potential effects of CeO_2_ NPs on agricultural plants.

## Data Availability

The data presented in this study are available in the Supplementary Material.

## Supplementary Materials

The following supporting information can be downloaded at: https://www.mdpi.com/article/10.3390/molecules28041798/s1, Table S1: Observed and certified values of elemental concentrations in standard reference materials, GBW07602 and GBW07603 (bush branches and leaves); Table S2: Summary of multiple stepwise regression and indices parameters for predicting the dry weight of plant; Figure S1: Contents of soluble protein, proline, and total phenol contents in the corn kernels harvested from plants cultivated in CeO_2_ NPs/CeCl_3_-amended soil; Figure S2: Contents of soluble protein, fat, reducing sugar, lysine and vitamin E contents in the soybean seeds harvested from plants cultivated in CeO_2_ NPs/CeCl_3_-amended soil. Different letters stand for statistical differences between treatments at *p* ≤ 0.05; Figure S3: Contents of Ca, P, S, Fe, Cu, Mn and Mo in the corn kernels harvested from plants cultivated in CeO_2_ NPs/CeCl_3_-amended soil. Different letters stand for statistical differences between treatments at *p* ≤ 0.05; Figure S4: Contents of Ca, Fe and Zn in the soybean seeds harvested from plants cultivated in CeO_2_ NPs/CeCl_3_-amended soil.

## Sample Availability

Samples of the compounds CeO_2_ NPs and CeCl_3_·7H_2_O are available from the authors.
