# Supplementary material for: Effects of CeO2 Nanoparticles on Nutritional Quality of Two Crop Plants, Corn (Zea mays L.) and Soybean (Glycine max L.)
Source: Molecules. 2023 Feb 14;28(4):1798. doi: 10.3390/molecules28041798 (PMC9960106; doi:10.3390/molecules28041798)
Supplement: Supplementary file 1 [file molecules-28-01798-s001.zip › molecules-2105428-supplementary.pdf]

Supporting Information

Effects of CeO_2 Nanoparticles on Nutritional Quality of Two Crop Plants, Corn (Zea mays L.) and Soybean (*Glycine max* L.)

Xin Gui [1,2,†], Chaonan Dong[1,3,†], Shixian Fan [1,2], Chunlei Jiao [1], Zhuda Song [1], Jiaqi Shen [1], Yong Zhao [2], Xuanzhen Li [2], Fawen Zhang [2*], Yuhui Ma [1], Xiao He [1], Aijun Lin [3*], Zhiyong Zhang [1,4*]

Table S1. Observed and certified values of elemental concentrations in standard reference materials, GBW07602 and GBW07603 (Bush branches and leaves)

Elements	GBW07602		GBW07603	
	Certified Value	Observed Value	Certified Value	Observed Value
K（g/kg）	8.5±0.5	18±2.3	9.2±1	10±0.7
Ca（g/kg）	22.2±1.3	21±0.5	16.8±1.1	16.3±2.3
S（g/kg）	3.20±0.30	4.1±8.1	7.3±0.6	6.6±0.8
P（g/kg）	0.83±0.04	0.8±0.07	1.00±0.04	0.93±0.03
Fe (mg/kg)	1020±67	863±29	1070±57	868.6±23
Mn (mg/kg)	58±6	60.28±1.91	61±5	66.69±5.69
Mo (mg/kg)	0.26±0.04	0.33±0.02	0.28±0.05	0.33±0.04
Cu (mg/kg)	5.20±0.50	4.95±0.22	6.6±0.8	6.11±0.45
Zn (mg/kg)	20.6±2.2	24.8±3.75	55±4	53.1±5.5
Ce (mg/kg)	2.40±0.30	3.05±0.97	2.2±0.1	2.92±0.28

Experimental values are presented as mean of 3 replicates with the corresponding standard deviation.

Table S2. Summary of multiple stepwise regression and indices parameters for predicting dry weight of plant

Dependent Variable（DV）	Fitted equation	R^2%	R^2-adj%	p-value	Durbin-Waston
Dry weight(corn kernels)	$y=17.93-0.003x_2$	37.3	34.3	0.002	1.288
Dry weight(soybean seed)	$y=0.443+0.151x_8$	22.3	19.2	0.013	2.170

R_2 %: coefficient of determination; R_2 -adj: adjusted coefficient determination. * and ** significant at the 0.05 and 0.01 level respectively.

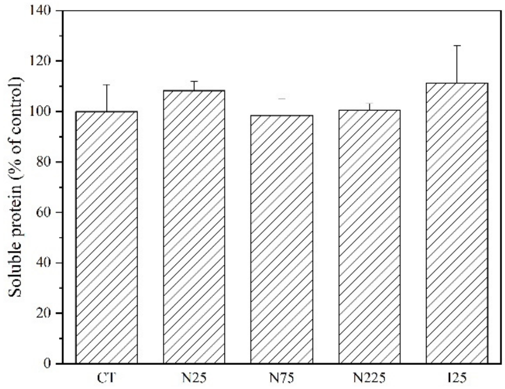

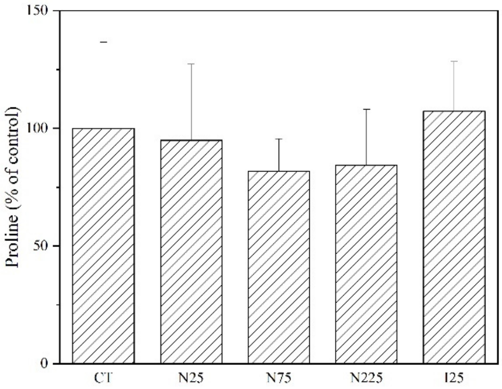

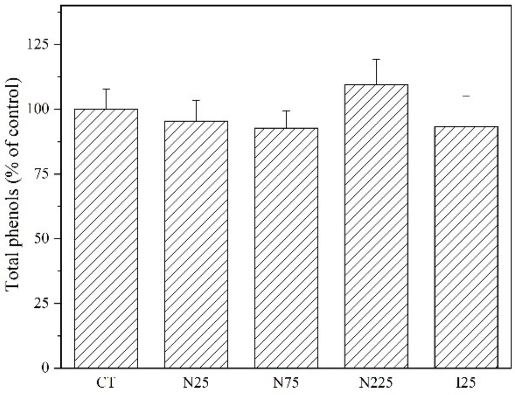

Figure S1. Contents of soluble protein, proline and total phenol contents in the corn kernels harvested from plants cultivated in CeO_2 NPs/$CeCl_3$-amended soil.

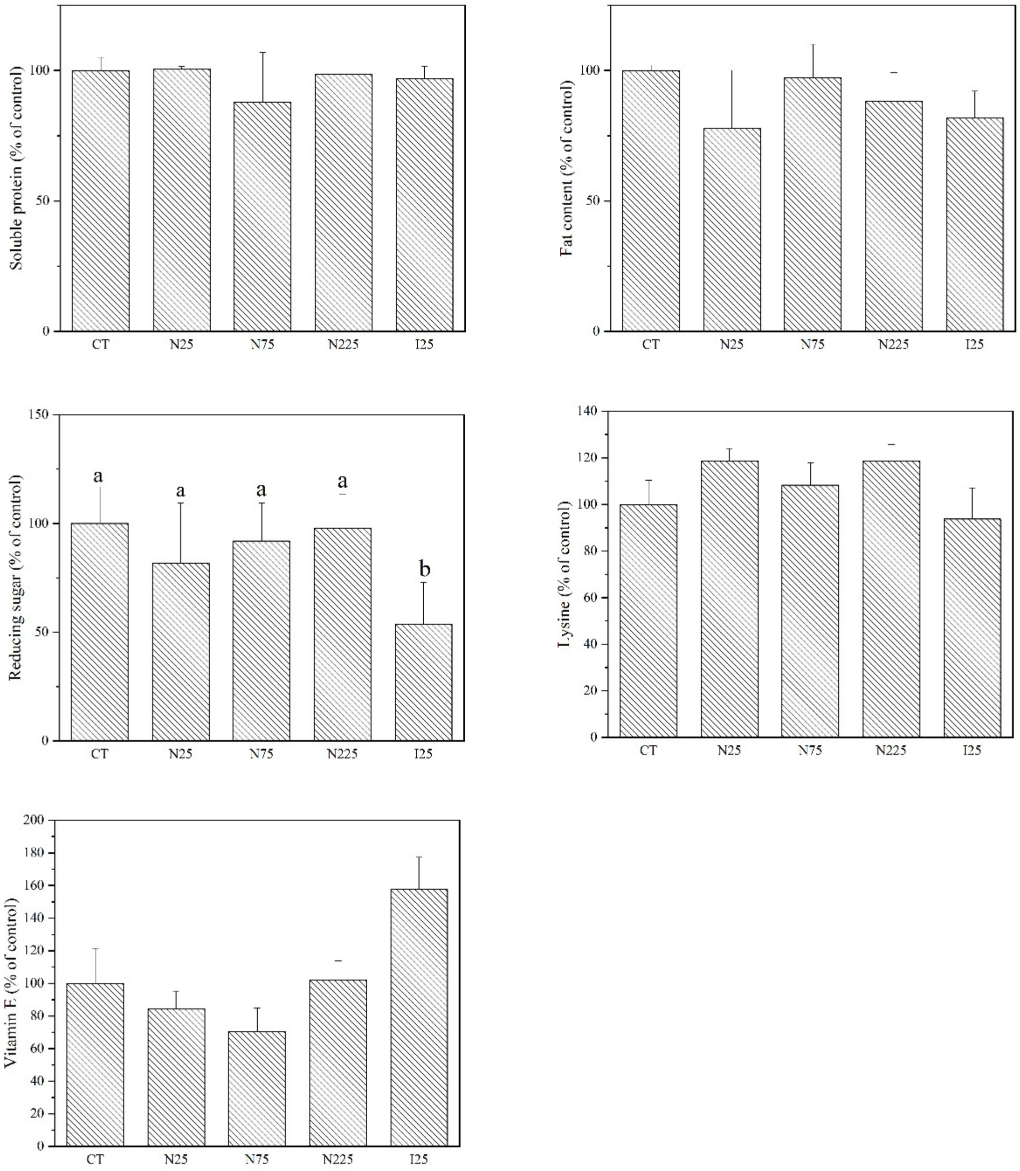

Figure S2. Contents of soluble protein, fat, reducing sugar, lysine and vitamin E contents in the soybean seeds harvested from plants cultivated in CeO_2 NPs/$CeCl_3$-amended soil. Different letters stand for statistical differences between treatments at $P \leq 0.05$.

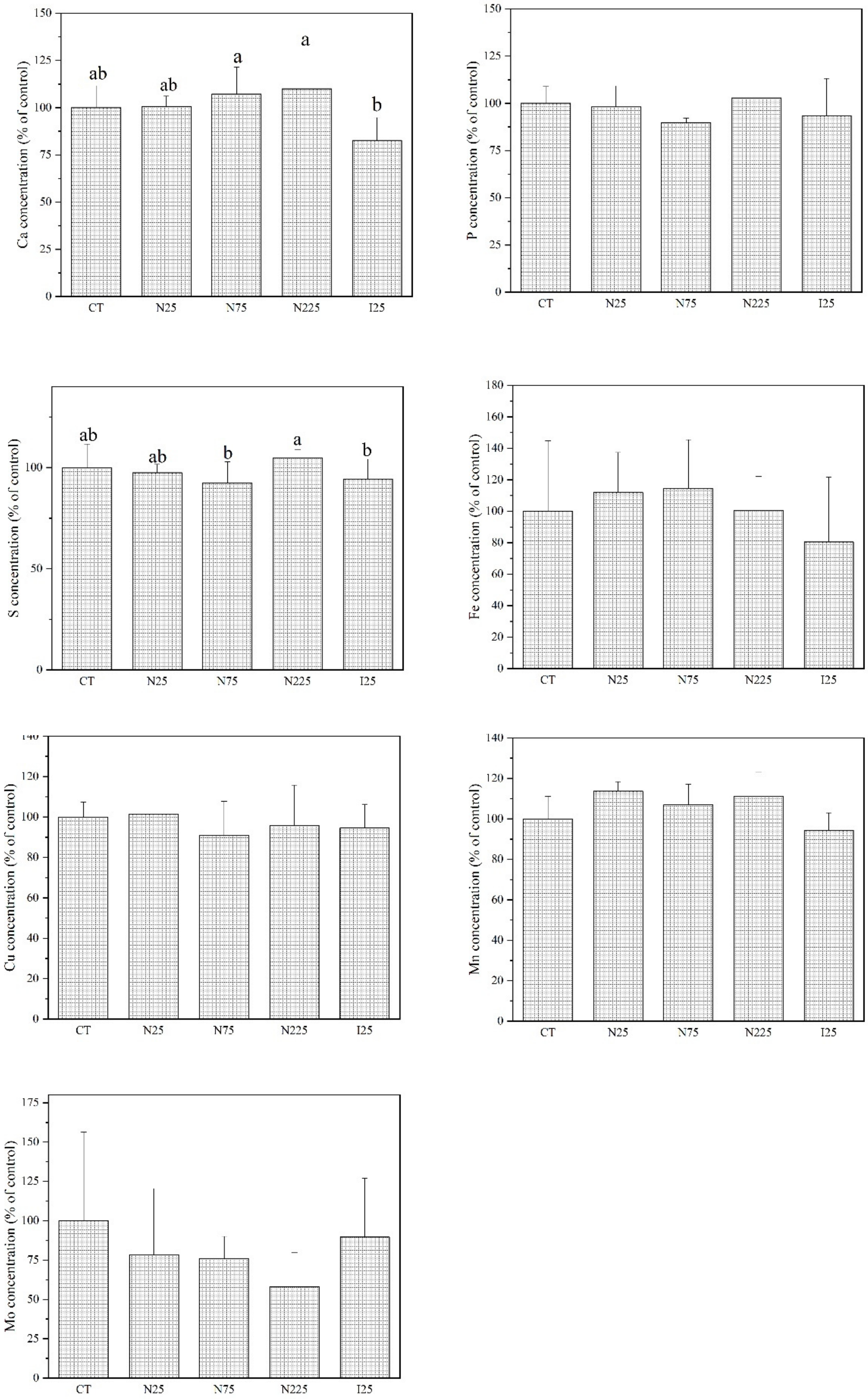

Figure S3. Contents of Ca, P, S, Fe, Cu, Mn and Mo in the corn kernels harvested from plants cultivated in CeO_2 NPs/$CeCl_3$-amended soil. Different letters stand for statistical differences between treatments at $P \leqslant 0.05$.

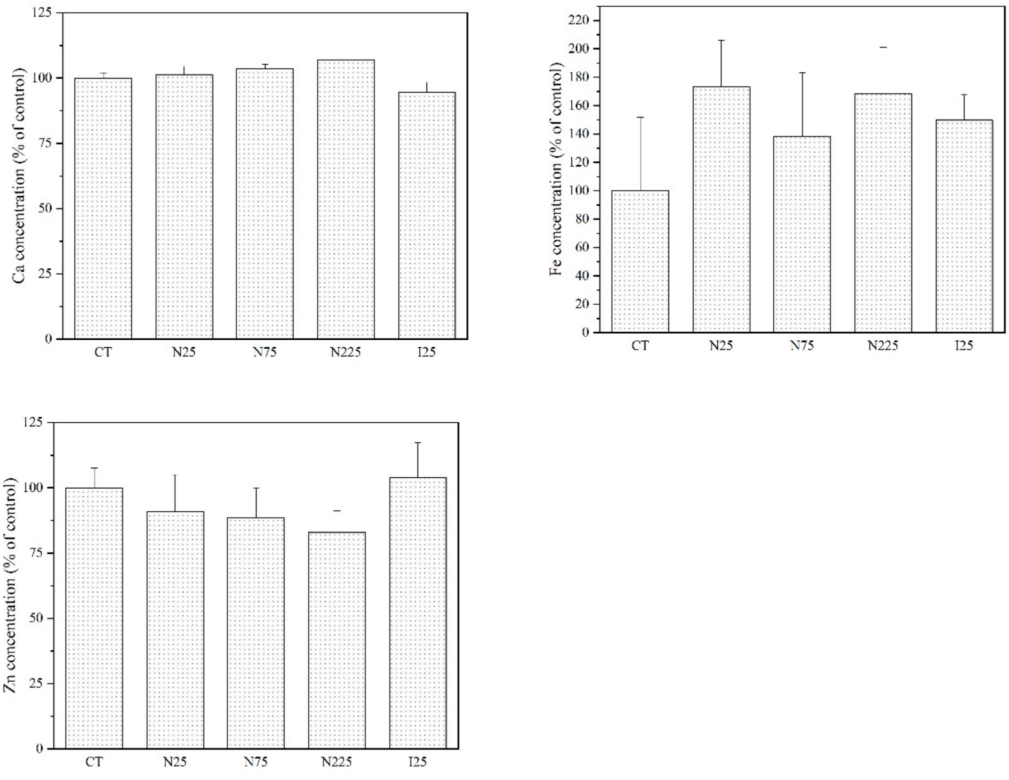

Figure S4. Contents of Ca, Fe and Zn in the soybean seeds harvested from plants cultivated in CeO_2 NPs/$CeCl_3$-amended soil.
